# Influence of Electrical Field Collector Positioning and Motion Scheme on Electrospun Bifurcated Vascular Graft Membranes

**DOI:** 10.3390/ma12132123

**Published:** 2019-07-02

**Authors:** Raquel Tejeda-Alejandre, Jan A. Lammel-Lindemann, Hernan Lara-Padilla, David Dean, Ciro A. Rodriguez

**Affiliations:** 1 Tecnologico de Monterrey, Escuela de Ingeniería y Ciencias, Monterrey, N.L. 64849, Mexico; 2 Laboratorio Nacional de Manufactura Aditiva y Digital (MADIT), Apodaca, N.L. 66629, Mexico; 3 Department of Plastic and Reconstructive Surgery, The Ohio State University, Columbus, OH 43210, USA; 4 Department of Surgery, Houston Methodist Hospital, Houston, TX 77030, USA; 5 Departamento de Ciencias de la Energía y Mecánica, Universidad de las Fuerzas Armadas ESPE, Sangolquí 171-5-231B, Ecuador

**Keywords:** additive manufacturing, electrospinning, tissue engineering, bifurcated vascular grafts, Y-grafts

## Abstract

Currently, electrospinning membranes for vascular graft applications has been limited, due to random fiber alignment, to use in mandrel-spun, straight tubular shapes. However, straight, circular tubes with constant diameters are rare in the body. This study presents a method to fabricate curved, non-circular, and bifurcated vascular grafts based on electrospinning. In order to create a system capable of electrospinning membranes to meet specific patient needs, this study focused on characterizing the influence of fiber source, electrical field collector position (inside vs. outside the mandrel), and the motion scheme of the mandrel (rotation vs. rotation and tilting) on the vascular graft membrane morphology and mechanical properties. Given the extensive use of poly(ε-caprolactone) (PCL) in tubular vascular graft membranes, the same material was used here to facilitate a comparison. Our results showed that the best morphology was obtained using orthogonal sources and collector positioning, and a well-timed rotation and tilting motion scheme. In terms of mechanical properties, our bifurcated vascular graft membranes showed burst pressure comparable to that of tubular vascular graft membranes previously reported, with values up to 5126 mmHg. However, the suture retention strength shown by the bifurcated vascular graft membranes was less than desired, not clinically viable values. Process improvements are being contemplated to introduce these devices into the clinical range.

## 1. Introduction

### 1.1. Justification

The generation of tubular membranes for vascular applications is commonly performed via electrospinning, as the basic manufacturing process for a resorbable or inert inner membrane. However, producing vascular graft devices with complex geometries is challenging. The use of resorbable materials for a curving, bifurcated graft is potentially beneficial to pediatric patients with congenital heart defects as well as pancreatitis patients undergoing resection of the pancreaticoduodenal junction. 

Congenital cardiac anomalies are the most common birth defect, affecting nearly 1% of all live births, with approximately 25% of patients with congenital heart disease having critical defects. The most common critical congenital heart defects are ventricular and atrial septal defects, which are two of a limited number of defects which may be monitored in certain patients [1,2]. With or without monitoring, congenital heart defects are the most common cause of newborn death. These defects require surgical or transcatheter intervention before one year of age. Technology and science have contributed to the production of new solutions and improved patient outcomes [3]. 

**Figure 1:**
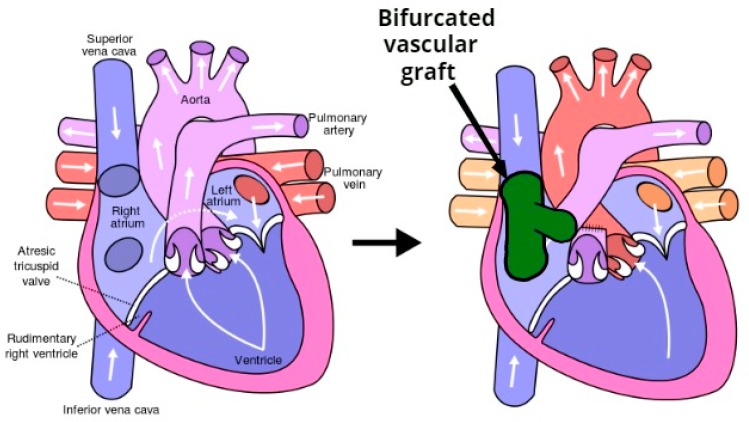
The Fontan procedure reconnects the inferior vena cava and superior vena cava to the pulmonary arteries avoiding the right ventricle.

A significant percent of the morbidity and mortality of pediatric cardiac surgery arises from the fact that the inert synthetic conduits and patches frequently used to repair congenital defects are susceptible to thromboembolism, stenosis, ectopic calcification, and infection [1]. Tissue engineering provides a potential solution to this problem. An implanted biodegradable vascular graft membrane degrades over time and is replaced with autologous vascular tissue that can repair, remodel, and even grow with the patient. The most important obstacle to performing bypass grafts in infants and children is the small caliber of corresponding vessels [4]. The Fontan surgical procedure (see Figure 1) is used for children with univentricular hearts and utilizes an oversized graft, which allows the child to “grow into” it. An ideal tissue-engineered vascular graft would be antithrombotic and nonimmunogenic, with an internal surface rapidly endothelialized. Rapid endothelialization is the best way to ensure thrombosis does not occur at the graft/host junction or on the graft itself.

Vascular graft biomechanical integrity, both initially and over time if tissue is to form, requires a durable structure with burst strength (i.e., biomechanical properties) akin to that of native vessels. This structural integrity is usually accomplished via a strong, but non-resorbable, outer layer, most often formed from expanded Polytetrafluoroethylene (ePTFE, i.e., Gore-Tex^®^) and polyethylene terephthalate (PET, or Dacron^®^) and polyurethane (PU) [5,6]. These grafts do not have growth capacity [4,7]. Thus, the ideal tissue engineering vascular grafts (TEVG) would be fully biodegradable, thereby obviating the need for risky serial reoperation to upsize the graft in response to somatic overgrowth [2]. There is a need for small-diameter vascular grafts for patients affected by pediatric congenital anomalies, vasospasm, limited length, and poor quality cardiac function.

**Figure 2:**
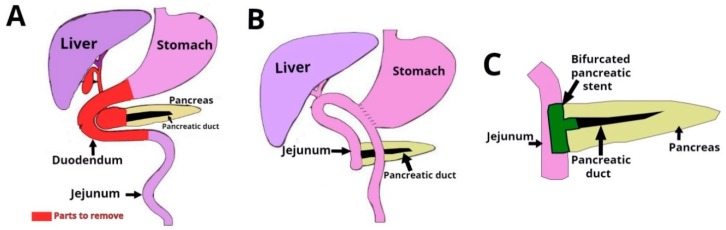
Pancreaticoduodenectomy (PD) or Whipple is a surgical procedure performed to remove cancerous tumors from the pancreas. The technique consists of the extirpation of a section of the stomach, the first and second portions of duodenum, the head of the head of the pancreas, the common bile duct, and the gallbladder.

The problems encountered in the design of vascular grafts are commonly encountered in pancreaticoduodenectomy (PD) procedures, the most common of which is the Whipple procedure (see Figure 2). While previously associated with morbidity and mortality rates that were considered prohibitive, the Whipple procedure is currently associated with postoperative mortality as low as 1% in high-volume medical centers. Several studies have reported the use of PD stents to decrease postoperative pancreatic leak and fistula from the cut surface of the distal pancreas [4]. 

The use of metal stents for drainage of pancreatic fluid collections is associated with improved clinical success, fewer adverse events and reduced bleeding compared to plastic stents. Plastic stents have substantially smaller lumens than metal stents leaving them more susceptible to blockage or occlusion [8]. In many cases, after healing, the stent is removed. 

Bifurcated grafts with a complex shape based on resorbable materials with tunable resorption rate and appropriate mechanical properties would be extremely useful in surgical operations such as the Fontan procedure for heart and the Whipple procedure for pancreas. This study focused on the development of this kind of next generation vascular graft. 

### 1.2. Related Work

Synthetic materials, such as poly(ε-caprolactone) (PCL), have been extensively employed in the research of vascular graft tissue engineering [3,5] because of its excellent biocompatibility, bioactivity, non-toxicity and, in some configurations, high elasticity and degradability. However, the long degradation period of pure PCL, usually more than 18 months in vivo, could act as a barrier to tissue regeneration if the healing window is missed. PCL is resistant to degradation because of its hydrophobic nature and high level of crystallinity [2,3]. On the other hand, if it fails mechanically before it resorbs, small, broken-up pieces can become distributed throughout the regeneration site, further preventing adequate tissue regeneration.

One of the principle tissue engineered vascular graft perquisites is that the grafts have similar mechanical properties to the native tissue at the placement site, before tissue regeneration and remodeling has taken place. A mechanical mismatch is acknowledged as a key determinant in the loss of long-term patency, resulting in aneurysm formation and implant failure [5]. Different approaches and techniques have been explored to produce a clinically viable vascular graft. These can be classified into different categories: vascular graft membrane-based, self-assembly processes [6], 3D printing, solvent casting, phase separation spinning, and electrospinning [9]. However, electrospinning is the most widely studied and used for the fabrication of vascular grafts.

The electrospinning process uses an electric field to direct a jet of polymer solution from a syringe’s capillary tip toward a target for deposition [10,11]. Under the influence of a strong electrostatic field, charges are induced in the solution and the charged polymer is accelerated towards the grounded metal collector. At low electrostatic field strength the pendant drop emerging from the tip of the pipette is prevented from dripping due to the surface tension of the solution. It is, therefore, critical to be able to control the syringe’s pump speed in terms of the release of the polymer within the speed window. The window must be between insufficient to form a thread and dripping that overtakes the surface tension needed to engage the electrical field. As the intensity of the electric field is increased, the induced charges on the liquid surface repel each other and create shear stresses [12]. In the electrospinning of vascular graft membranes, fiber diameter, orientation, alignment (mandrels increase the possibility of alignment), and pore size have a significant impact on the final functionality of the vascular conduit [13]. According to the standard procedures for setting up a basic electrospinning process [14], once the polymer is selected, the literature recommends two principles to select the solvent: (a) the polymer should be completely soluble and (b) the boiling point of the solvent should be moderate in order to allow evaporation during the trajectory of the solution between the needle tip and the collector. Some of the most common solvents used in combination with PCL are hexafluoropropanol (HFP), chloroform, acetone and dimethylformamide (DMF) [15]. 

The most important point in vascular tissue engineering is simulating the native tri-layered structure and recovering vascular function on placement and throughout the regenerative process. As previously noted, electrospinning methods have been primarily used to generate the internal membrane. The diameter and orientation of fibers and the pore geometry and permeability are essential to rapid cell attachment and endothelialization. It is equally important to develop a mechanically long-lasting and functionally sustainable tissue-engineered vascular graft for clinical application in the outer layers [16]. Reports of earlier relevant studies (see Table 1) show the careful consideration of vascular graft morphology and mechanical characteristics. Techniques like electrospinning allow the construction of tubular vascular graft membranes through a rotating mandrel, acting as an internal collector to attract the fibers. 

Efforts to emulate complex geometries such as bifurcations date back to the 1970s, when a graft made out of Dacron fiber was used as an arterial bypass graft in 135 patients [25]. Later, a Y-graft tailored from a bovine pericardium was created from two tubes of pericardium [26]. These bovine grafts were closer in size to the human aorta and common iliac arteries than other available standard bifurcated grafts. 

Most progress in tissue engineered vascular grafts has been concentrated on tubular shapes (see Table 1). More recently, tissue engineering approaches have used different synthetic materials to create customizable bifurcated grafts or a combination of different technologies, such as 3D printing, electrospinning, and custom-designed mandrels with cardiovascular magnetic resonance imaging (MRI) datasets from different patients [27], aiming to emulate the original tissue, in terms of mechanical and morphological characteristics. 

Despite the progress, there is still a need for a bifurcated vascular graft based on resorbable materials, and with appropriate mechanical properties. This study focused on the performance of these types of complex shapes and mechanical properties.

### 1.3. Objective

In this study we tested the bifurcated vascular graft membranes that we fabricated via electrospinning. Through several iterations of design–prototype–test we refined a manufacturing system with a custom-made positioning mechanism and a custom collector (patent pending [28]). In this study the objective was to explore the influence of collector placement and motion scheme on graft membrane external shape, fiber diameter, and mechanical properties. The collector was connected both inside and outside the mandrel on different configurations. The motion schemes used include: (a) rotation vs. (b) the combination of rotation and longitudinal movement. Bifurcated vascular grafts were characterized via both morphology measurements and mechanical testing.

## 2. Materials and Methods

### 2.1. Fabrication Process

#### 2.1.1. Positioning the Mechanism

**Figure 3:**
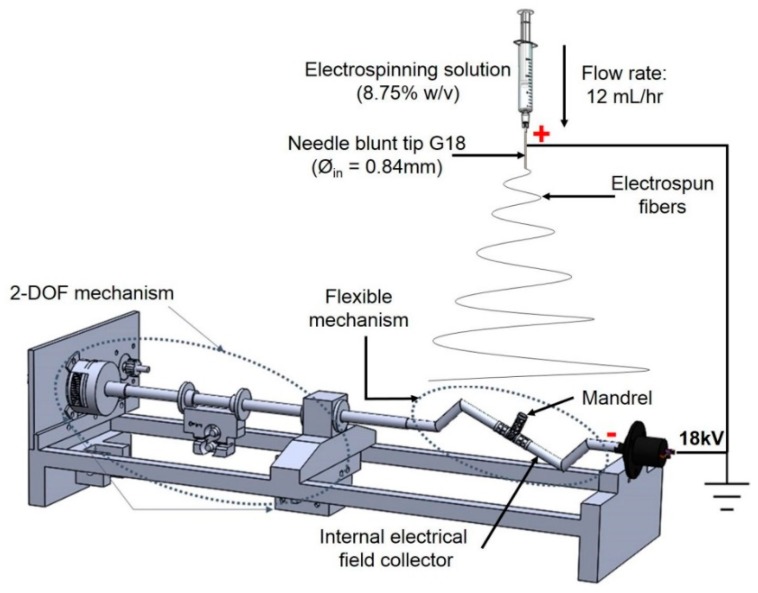
Two degrees of freedom (2-DOF) mechanism used for bifurcated vascular graft membrane.

The proof-of-concept for the type of positioning mechanism used in this study was reported by [29] (see Figure 3). It was based on two degrees of freedom (2-DOF) with rotational and longitudinal motion that tilted the mandrel in order to create different relative angles between the mandrel and the electrospinning needle.

#### 2.1.2. Mandrel and Electrical Field Collector

**Figure 4:**
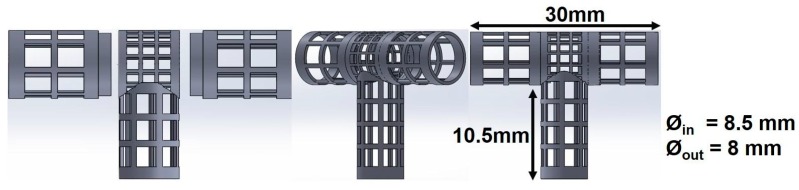
Dimensions of 3D printed bifurcating vascular graft mandrel.

**Figure 5:**
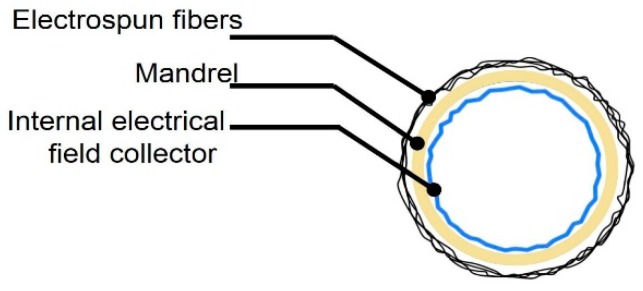
Vascular graft membrane cross-section during electrospinning process, showing location of internal electrical field collector.

The mandrel design was created as a parametric model using SolidWorks 2018 (Dassault Systèmes, Waltham, MA, USA), with a bifurcated tubular shape, as shown in Figure 4. To facilitate the deposition of fibers that fully coat the mandrel, an internal collector was designed (see Figure 5). The aim was to transform the flexible mechanism where the mandrel is mounted into a collector that fills its internal space entirely, and evenly, with a layer of conductive material.

The mandrels were fabricated via continuous digital light processing (cDLP) with a 3D printing projector system, which created solid objects by photopolymerization of a liquid polymer resin [30]. A high-resolution EnvisionTec Perfactory 3 Multilens machine cDLP was used to 3D print, with E-Shell 300 polymer resin (EnvisionTEC, Dearborn, MI, USA), the mandrels used in this study. 

**Figure 6:**
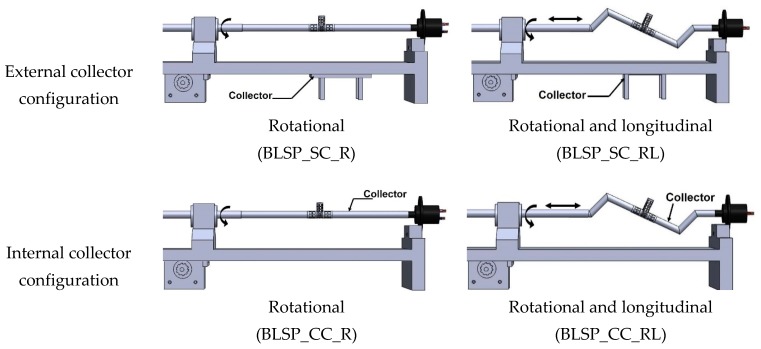
Schematic illustration of electrical field collector configuration and mandrel motion scheme.

Electrospinning was done under four different sets of process conditions (see Table 2), including external vs. internal electrical field collector and two types of motion schemes for the mandrel positioning relative to the source of fibers (needle). The collector configuration and motion scheme are illustrated in Figure 6. A total of 6 replications were used for each combination of process parameters.

#### 2.1.3. Electrospinning Process

Polycaprolactone (PCL) pellets (Polysciences Inc, Warrington, PA, USA) with a molecular number (Mn) of 80,000 g·mol^−1^ were used. Acetone was selected as the solvent and was a non-variable parameter. A PCL solution was prepared by dissolving 8.75 wt % of PCL and acetone. The electrospinning was conducted at 18 kV (voltage source model ESP20P-5W, Gamma High Voltage Research Inc., Ormond Beach, FL, USA) with a flow rate of 12 mL·h^−1^ using a syringe pump, Harvard model PHD2000 (Harvard Apparatus, Holliston, MA, USA), and a distance of 7–10 cm between the collector and the tip of the needle. The influence of this factor was not evaluated in this study. This approach was used to form a mat of fibers on the mandrel’s surface (see Figure 4).

### 2.2. Characterization of Vascular Graft Membrane Morphology

The following measurements were used to characterize the samples: a) fiber diameter, b) corner profile fidelity (F_CP_ [%]) to determine how the electrospun fibers adhered to the shape of mandrel, c) membrane thickness (t[μm]) of the resultant vascular graft membrane, and d) burst pressure reached by the bifurcated vascular graft membrane. The vascular graft membrane was divided into regions to analyze how the electrospun fibers were distributed over the mandrel. The procedures are detailed next.

#### 2.2.1. Fiber Diameter and Alignment

A scanning electron microscope (SEM) EVOMA25 (Carl Zeiss, Oberkochen, Germany) was used to characterize the vascular graft membrane morphology. The coating procedure was conducted using gold. Images were acquired using conventional SEM operating at an accelerated voltage of 5 kV with a working distance of 9 mm. The images were analyzed using ImageJ software (version 1.44p, U.S. National Institutes of Health, Bethesda, MD, USA) to determine the diameter of fibers and alignment. Fiber diameter measurements were conducted at 200 random positions. Distribution and average diameter were computed and reported. The alignment analysis was achieved using ImageJ software.

#### 2.2.2. Corner Profile Fidelity 

**Figure 7:**
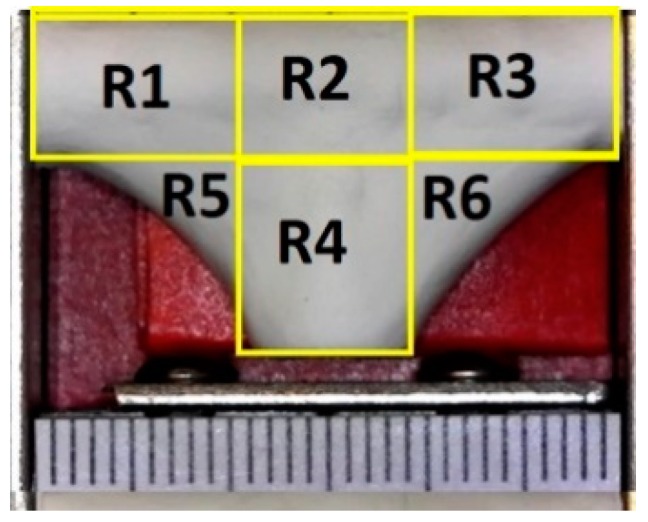
Definition of measurement regions for corner profile fidelity—each mark on the scale is 1 mm.

In order to characterize how closely the vascular graft membrane followed (adhered to) the surface of the mandrel, we defined the following indicator: corner profile fidelity (F_CP_). This indicator was defined as the percentage of projected area without fibers, relative to the total area in a picture taken from a fixed angle on regions R5 and R6, as defined in Figure 7. Therefore, a higher F_CP_ indicated a better vascular graft membrane that fits more closely to the surface of the mandrel shape, and this translated to a bifurcated shape of better quality.

**Figure 8:**
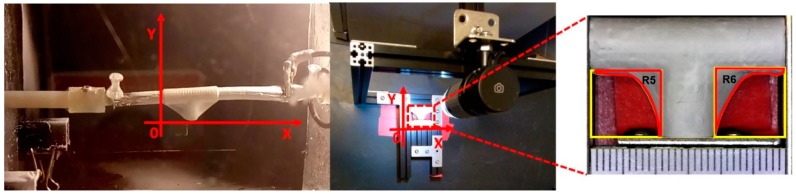
The position to observe the sample and analyze F_CP_ (%) related to the direction it was electrospun to characterize how the electrospun material was deposited over the mandrel.

**Figure 9:**
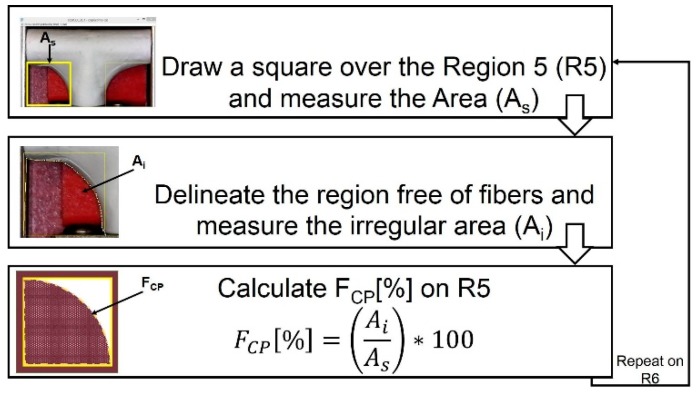
Measuring process to calculate corner profile fidelity F_CP_ (%) on regions R5 and R6 of the vascular graft membrane with ImageJ software.

An optical microscope USB2-MICRO-250X (Plugable Technologies, Redmond, WA, USA), with 0x–250x magnification range, was used to visualize the corner profile’s fidelity to the mandrel surface, F_CP_ [%]. Images were then measured in ImageJ. The samples were photographed in the same position they were in when extracted from the positioning mechanism (see Figure 8). Based on the “measurement area” tool available on ImageJ, F_CP_ [%] was determined. The results are expressed as the mean ± standard error. The corner profile fidelity was measured while the mandrel was still inside. The procedure was as follows: a) draw a square around region R5 with “rectangle” tool on ImageJ to measure the resulting area to establish the reference total area (A_s_); b) delineate the area free of fibers, inside the previously drawn rectangle, and register the measurement (A_i_) with “polygon selections” tool; and c) compute F_CP_ [%] on R5 as $F_{CP}\left[\%\right]=\left(\frac{A_{i}}{A_{s}}\right)*100$. The same process was repeated for region R6. The whole measurement protocol is summarized in Figure 9. 

#### 2.2.3. Thickness Measurement

**Figure 10:**
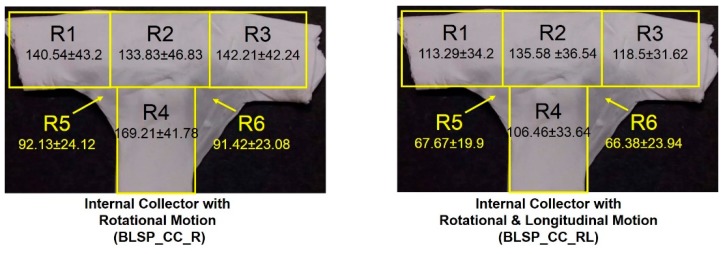
Regions for membrane thickness measurement.

After removal of the mandrel, membrane thickness (t) in each region was measured with a digital micrometer (IP65 coolant proof, Mitutoyo, Aurora, IL, USA) with a resolution of ±1 μm (see Figure 10). For consistency of these measurements, a fixture was constructed to hold the micrometer and great care was taken to not break the vascular graft membranes. 

### 2.3. Characterization of Mechanical Properties 

#### 2.3.1. Burst Pressure Test

**Figure 11:**
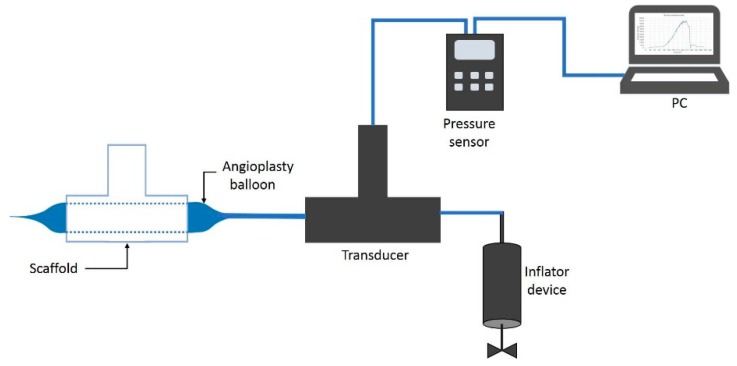
Burst pressure test system.

Using the bifurcated electrospun vascular graft membranes and an angioplasty balloon, pressurization to burst was achieved and the pressure at bursting was measured (see Figure 11). The measurements were conducted on all electrospun bifurcated vascular graft membranes (30 mm length). The burst set-up began with an angioplasty balloon (Cordis, Co., Hialeah, FL, USA) being inserted into the vascular graft membrane. The membrane had been hydrated for 6 h with phosphate buffered saline (PBS) at ambient temperature prior to this test. An Encore™ 20 Inflator (Boston Scientific, Marlborough, MA, USA) was used to expand the balloon. Pressure was registered by a digital pressure meter (Wide Range Pressure Meter 840065, Sper Scientific, Scottsdale, AZ, USA), together with a LabVEW.vi system. The inflator, with the mounted vascular graft membrane, was filled with distilled water at room temperature, while the LabView routine recorded the pressure. The burst pressure was defined as the highest pressure reached before failure.

#### 2.3.2. Suture Retention Strength

**Figure 12:**
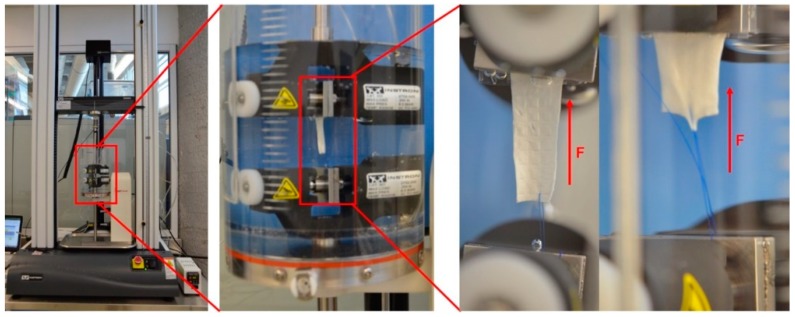
Set up used for mechanical characterization.

For this test, samples were cut out of the bifurcated membrane to generate a flat rectangular shape. Therefore, the thickness of these samples corresponded to the average thickness in regions R1, R2 and R3. Characterization, to determine the force necessary to pull a suture from the vascular graft membrane, was carried out using a universal testing machine Instron 3365 (Norwood, Massachusetts, USA), according to the International ISO 7198:2016 for Cardiovascular implants and extracorporeal systems -- Vascular prostheses -- Tubular vascular grafts and vascular patches, with a 5 N load cell under physiological conditions [31] and a temperature controlled bath in PBS at 37 °C (BioPuls Temperature Controlled Bath 3130-100), as shown in Figure 12. A Prolene 5–0 (Ethicon, Inc, Bridgewater, NJ, USA) suture was pierced through the wall of the vascular graft membrane 2.0 mm away from the edge and tied to form a loop. The thread was passed through the vascular graft membrane by means of the needle provided. The non-sutured end of the vascular graft membrane and the suture loop were each attached to separate clamps in the universal testing machine and the suture was pulled at 5 mm per minute. The suture point was centered with respect to the specimen width and its distance from the clamp [32]. 

### 2.4. Cytotoxicity Assay

To assess the in vitro cytotoxicity of the produced mats, the International ISO 10993-5 for Biological evaluation of medical devices was followed. Samples of PCL mats were soaked in 70% ethanol for 30 min, followed by washing, twice, in sterile PBS. Then, the samples were exposed to UV light for 15 min, 30 min prior to cell seeding.

Human fibroblasts (Detroit 548 ATCC, CCL-116, American Type Culture Collection, Rockville, MD, USA) were placed into sterile of 96-well plates with low glucose Dulbecco’s Modified Eagle’s Medium (DMEM, GIBCO, Invitrogen, Grand Island, NY, USA) supplemented with 10% FBS (Laboratorios Microlab, Mexico City, Mexico) and 1% penicillin–streptomycin (Sigma, St. Louis, MO, USA). The cultures were kept at 37 °C and 5% CO_2_ in an incubator (Sanyo, MCO-19AIC, (UV), Moriguchi, Japan) for 24 h to allow attachment. The culture medium was replaced every day and the cells were trypsinized upon reaching a confluence of 50%. Cell proliferation was assessed using MTT assay. 

This method was based on the conversion of MTT (3-(4,5-Dimethylthiazol-2-yl)-2,5-diphenyltetrazolium bromide) into formazan, which determined the mitochondrial activity. Fibroblasts were seeded, in triplicate, in 96-well culture microtiter plates at a density of 3 × 10^3^. Next, 100 µL of medium without cells and 100 µL of 1% Triton X-100 in PBS were added to the wells and were used as negative and positive controls, respectively. The microtiter plates were incubated for 96 h in an incubator with 5% CO2 at 37 °C. After incubation, 20 µL of MTT were added to the test wells and allowed to react for 6 h at 37 °C and 5% CO_2_. Afterwards, the medium was discarded and replaced with 150 µL of dimethyl sulfoxide. After gently shaking for 10min, the optical density (OD) was determined by reading the absorbance at 500 nm on a Biotek Synergy 2 microplate reader (Winooski, VT, USA). 

### 2.5. Statistical Analysis

GraphPad Prism Software Version 8.0 (San Diego, CA, USA) was used for statistical analysis. Data were expressed as mean ± standard deviation (SD). Univariate statistical comparisons were made with a Student’s t-test. Multivariate statistical analysis was carried out using ANOVA and Tukey’s tests. A p-value of p < 0.05 was considered statistically significant.

## 3. Results

### 3.1. Fabrication Process

**Figure 13:**
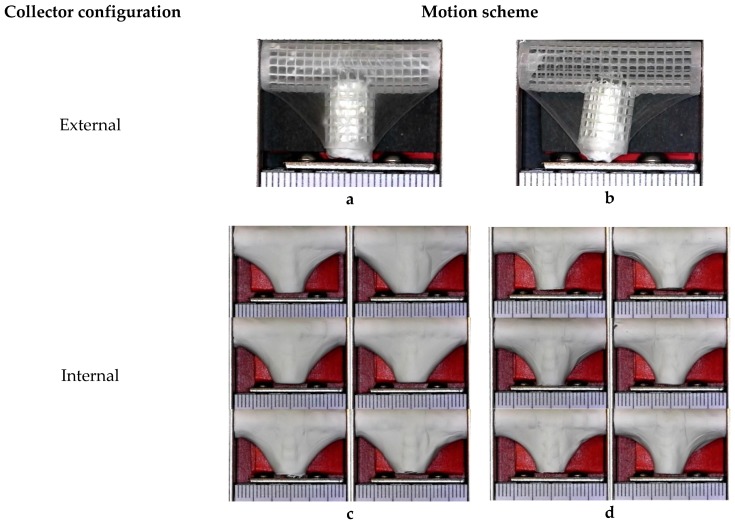
Overview of bifurcated vascular grafts obtained under different sets of process parameters. (**a**) Rotational (BLSP_SC_R); (**b**) Rotational and longitudinal (BLSP_SC_RL); (**c**) Rotational (BLSP_CC_R); (**d**) Rotational and longitudinal (BLSP_CC_RL).

Four groups of mandrels were electrospun under different conditions, as indicated in Table 2. Figure 13a,b shows that bifurcated vascular graft membranes were not viable when the electrical field collector was outside the mandrel. When the collector was placed inside the mandrel, it attracted a significant amount of fibers, as shown in Figure 13c,d.

### 3.2. Characterization of Vascular Graft Membrane Morphology

#### 3.2.1. Fiber Diameter and Alignment

**Figure 14:**
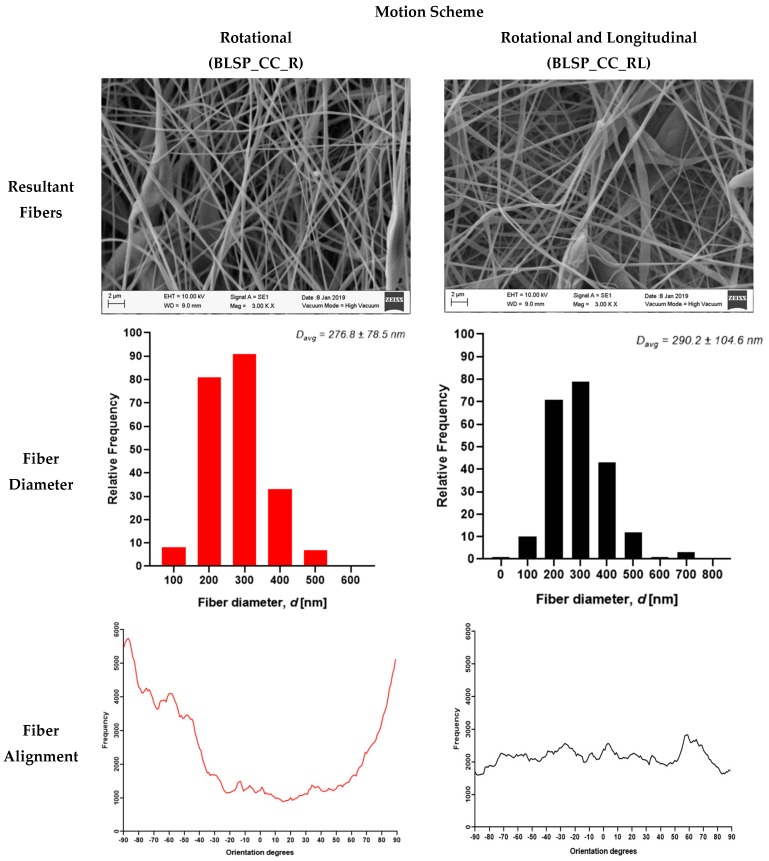
Electrospun fibers of the outer layer from bifurcated vascular graft membranes (SEM imaging).

After imaging and analysis, the mean observed fiber diameter for single axis rotation-only average fiber diameter was 290.2 ± 104.6 nm and 60° alignment fiber around 3000 measured fibers, while a motion scheme that involved rotational and longitudinal motion resulted in an average fiber diameter of 276.8 ± 78.5 nm and around –90°. Almost 6000 fibers were measured, as shown in Figure 14. 

#### 3.2.2. Corner Profile Fidelity (F_CP_)

**Figure 15:**
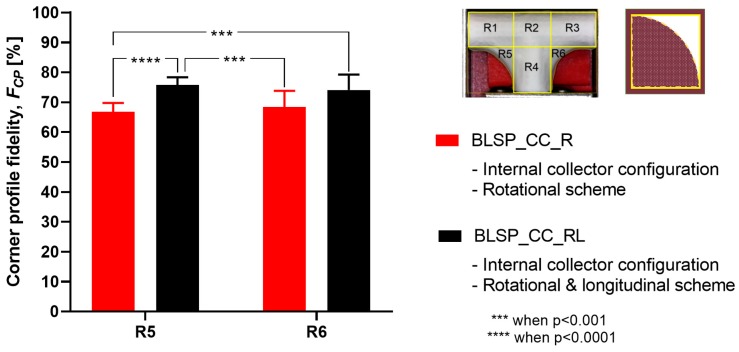
Average corner profile fidelity at regions R5 and R6. (A two-way ANOVA showed that the motion scheme with rotational and longitudinal approach increased the corner profile fidelity).

The goal was to obtain the highest F_CP_ to show that the electrospun membrane properly fit the given shape of the mandrel. There was no significant difference between region R5 and R6 (see Figure 15). However, it was clear that the combination of rotational and longitudinal motion on the mandrel produced a better fit of the membrane.

#### 3.2.3. Membrane Thickness Distribution

**Figure 16:**
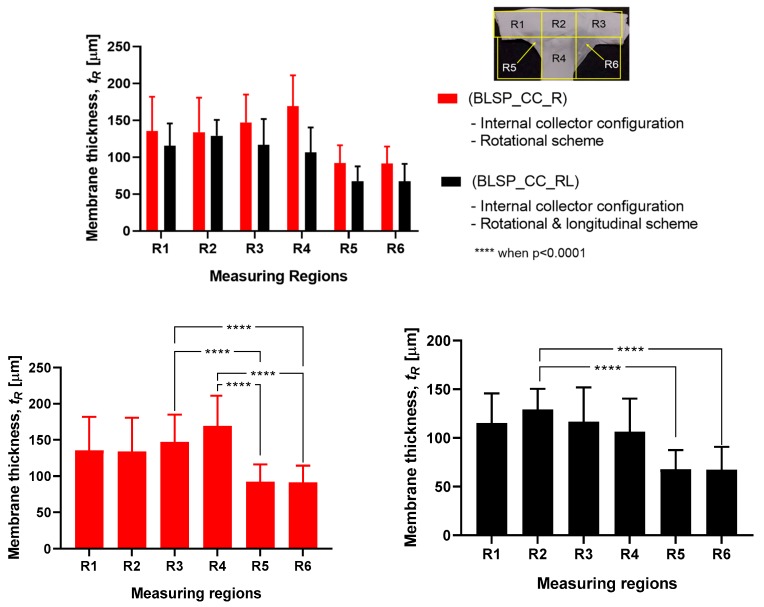
Membrane thickness distribution along the vascular graft membrane surface. (A two-way ANOVA was performed to determine any statistically significant differences between experiments.).

**Figure 17:**
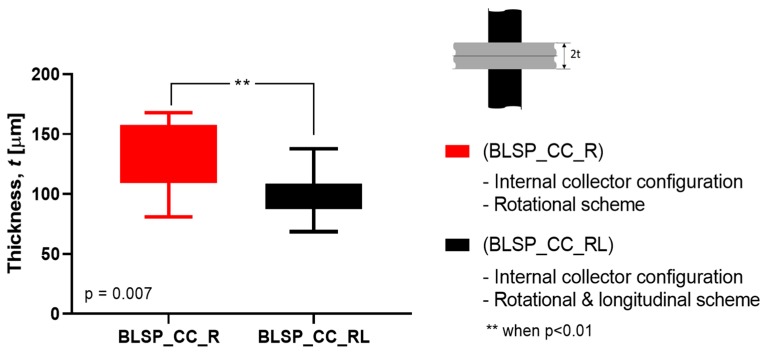
Average membrane thickness of the whole vascular graft considering all regions. (A Student’s t-test was performed to determine any statistically significant differences between experiments. All tests were carried out at a 95% confidence interval).

The vascular graft membrane thickness of 6 different regions was measured. Table 3 and Figure 16 show the membrane thickness per region. Figure 17 shows the average membrane thickness considering all regions.

The motion scheme for mandrel positioning influenced the average membrane thickness, where the rotational and longitudinal approach produced a thinner membrane. The motion scheme also clearly generated a different membrane thickness distribution among the different regions. Under rotational motion, R4 tended to have the thickest membrane (although not statistically significant when compared to R1 and R2). Under both motion schemes there was a statistically significant difference between R5 and R6 compared to at least one of the other regions. The fact that R5 and R6 had thinner membranes was thought to be due to their location at the corner of the mandrel, where deposition of electrospun fibers tended to be more difficult.

### 3.3. Characterization of Mechanical Properties

#### 3.3.1. Burst Pressure Strength

**Figure 18:**
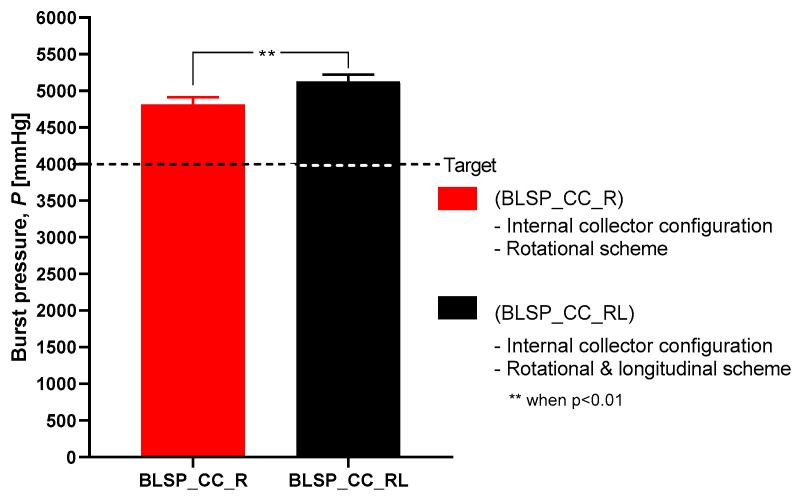
Burst pressure strength.

Figure 18 shows the observed results of the burst pressure test for both groups. Both met or exceeded the goal (4000 mmHg) according to [33]. These results suggested that burst pressure was not related to any difference in thickness of the two vascular graft membranes. In addition, the motion approach based on rotational and longitudinal motion produced a statistically significant increase in bust pressure strength, with an average of 5126 mmHg.

#### 3.3.2. Suture Retention Strength (SRS)

**Figure 19:**
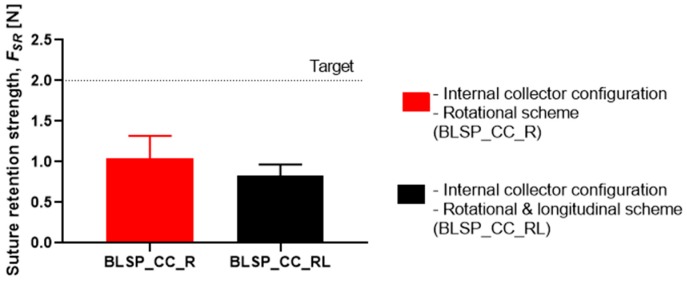
Suture retention strength. (A Student’s t-test was performed to determine any statistically significant differences between experiments. All tests were carried out at a 95% confidence interval and the results did not show statistically significant differences.)

**Figure 20:**
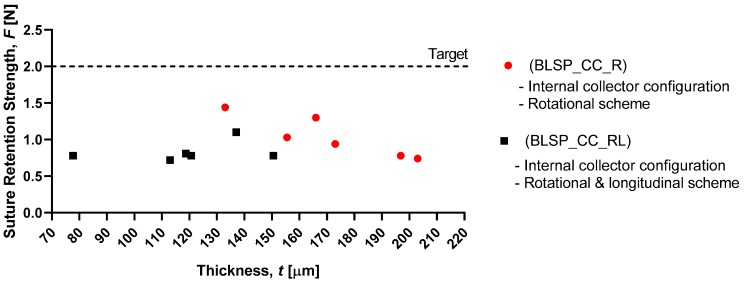
Membrane thickness vs. graft suture retention strength.

Figure 19 shows that the motion scheme did not influence the suture retention strength. There was no statistical difference between these conditions (rotational vs. rotational and longitudinal motion). The vascular graft membrane with the highest SRS was on the experiment BSLP_CC_R at 1.44 N, which meant 75% of the minimum clinical requirement [24,32,34]. More importantly, neither graft obtained the target strength. In addition, Figure 20 shows that while member thickness changed with motion scheme, thicker membranes did not produce an increase in suture retention strength. 

### 3.4. Cytotoxicity Assay

Our data revealed that all bifurcated scaffolds showed viability higher than 86%, which confirmed that PCL bifurcated scaffolds presented cytocompatibility (cell viability higher than 50%).

### 3.5. Summary of Results

Table 4 shows a summary of the experimental results. The motion scheme, by comparing only the rotation of the mandrel relative to the fiber source vs. rotation and tilting produced by the longitudinal movement, produced different effects on the response variables of interest. The application of rotational and longitudinal motion by the positioning mechanism produced statistically significant effects on the corner profile fidelity, burst pressure strength, and suture retention strength.

## 4. Discussion

The work presented here demonstrates that bifurcated vascular grafts can be constructed via electrospinning by combining an internal electrical field collector design and a 2-DOF positioning mechanism, for the mandrel. The addition of a second longitudinal axis for tilting of the mandrel improved the corner profile fidelity of the vascular graft membrane. However, it was not closely adherent in either case, suggesting that a more direct, perhaps robotically-assisted, approach to controlling needle tip orientation and mandrel surface was needed to ensure closer apposition. Alternatively, ejection pressure, mandrel speed, and/or needle tip distance to the mandrel may also improve apposition.

### 4.1. Characterization of Vascular Graft Membrane Morphology 

Vascular graft membrane morphology was evaluated through fiber diameter, corner profile fidelity (F_CP_), and membrane thickness. The average fiber diameter obtained in these experiments was 276.8 and 290.2 nm. These diameters were thinner but comparable to the low end of those previously reported, as shown in Table 1, where membrane thickness ranged between 0.4 and 900 μm. According to the literature, the diameter range of the self-expandable metal stents (SEMS) used as pancreatic stents is about 8–10 mm [35]. The bifurcated graft that we produced was within this range (internal diameter of 8 mm). The alignment obtained can be attributed to the motion scheme reported in the literature [36]. When high-speed profiles and rotational collectors were used, fiber orientation between –90° and +90° was expected. Based on the corner profile fidelity (F_CP_) indicator there was a clear benefit to having a combination of rotational and longitudinal motion. In the case of region R6, this benefit was not statistically significance. The need for better apposition of the vascular graft membrane to the mandrel, especially in regions R5 and R6, suggested that the relationship of speed, fiber alignment, and mechanical properties could be further explored, as suggested by Fukunishi et al. [17]. The study of membrane thickness showed that the rotational and longitudinal motion tended to lower this value (although no statistical significance was observed with the current data).

Previous studies demonstrated that PCL was a promising candidate for vascular grafts due to its bioactivity and nontoxicity [23,36]. In this study, the cytotoxicity assessment using MTT assay showed that the produced mats based on 3D printed mandrels provided a suitable surface to maintain adhesion of the seeded cells. The assay did not reveal whether fibroblasts penetrated the porous mats (see Figure 14). However it was assumed that the cells could infiltrate this type of fiber. The scope of the reported study was to obtain the necessary bifurcated morphology and mechanical properties for the proposed applications. Therefore, further studies are required to validate the proliferation of the seeded cells into the bifurcated grafts.

**Figure 21:**
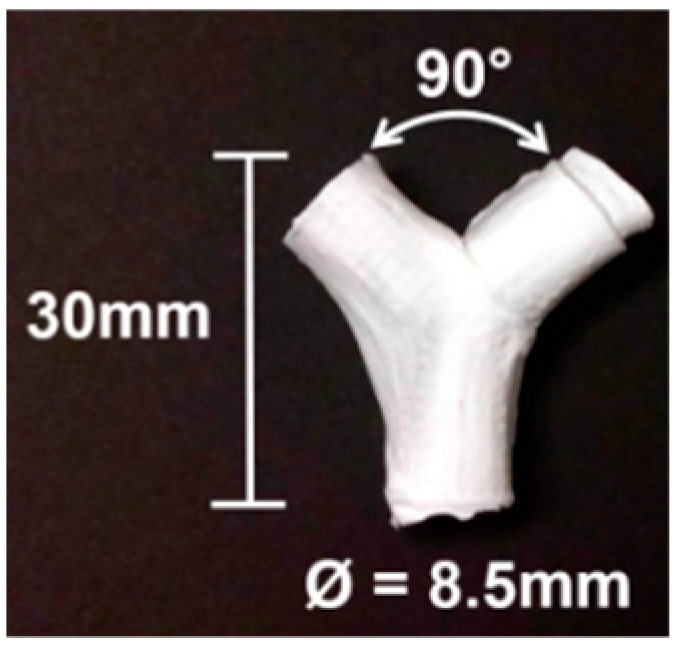
Vascular Y-graft produced by electrospinning, with a the 2-DOF positioning system and an internal electrical field collector approach.

This study focused on T-shaped bifurcated vascular grafts. Using the same approach as that based on the 2-DOF positioning system and internal electrical field collector design, it was possible to generate Y-shaped bifurcated vascular grafts, as shown in Figure 21. This kind of Y-graft was similar to those reported in related studies [27]. An improvement could be to construct a mandrel based on patient image studies and, therefore, generate patient-specific bifurcated vascular grafts with varying cross-sections and complex overall geometries. In this process F_CP_ was a relevant quantitative measurement of the quality of the constructed bifurcation structure. The objective continues to be to obtain an F_CP_ closer to 100%, to achieve patient-specific bifurcated shapes.

### 4.2. Characterization of Mechanical Properties

The burst pressure strength (BPS) tests showed that the proposed approach yielded acceptable results. For the rotational and longitudinal motion approach, an average BPS of 5126.6 ± 1074.57 mmHg was obtained for the bifurcated vascular grafts. This may have correlated with other measured characteristics such as fiber diameter. However, that variable was not studied. These levels of strength were comparable to those reported for straight tubular electrospun vascular graft membranes that did not change diameter (see Table 1). These values ranged between 985 and 4915 mmHg (except for the case of Kim et al. where an extremely thick membrane of 0.33 mm was tested [24]). Previously reported results for SRS suggest that this parameter was determined mostly by membrane thickness [37]. However, in the range of test conditions covered in this study, we did not find such a correlation. The SRS values for the bifurcated vascular graft membranes observed in the two groups in this study (1.04 N and 0.83 N) were not satisfactory according to the established target value (2 N based on [24,32,34]) and those shown in Table 1. Further process parameter optimization for apposition to the mandrel, increasing membrane thickness, or a change in materials could improve SRS, for potential vascular applications. In the case of the Whipple procedure, SRS was not as critical when compared to vascular applications. In that context, we expect that degradation kinetics under the influence of bile will be a challenge. 

### 4.3. Relationship between Process Parameters and Vascular Functional Specifications.

**Figure 22:**
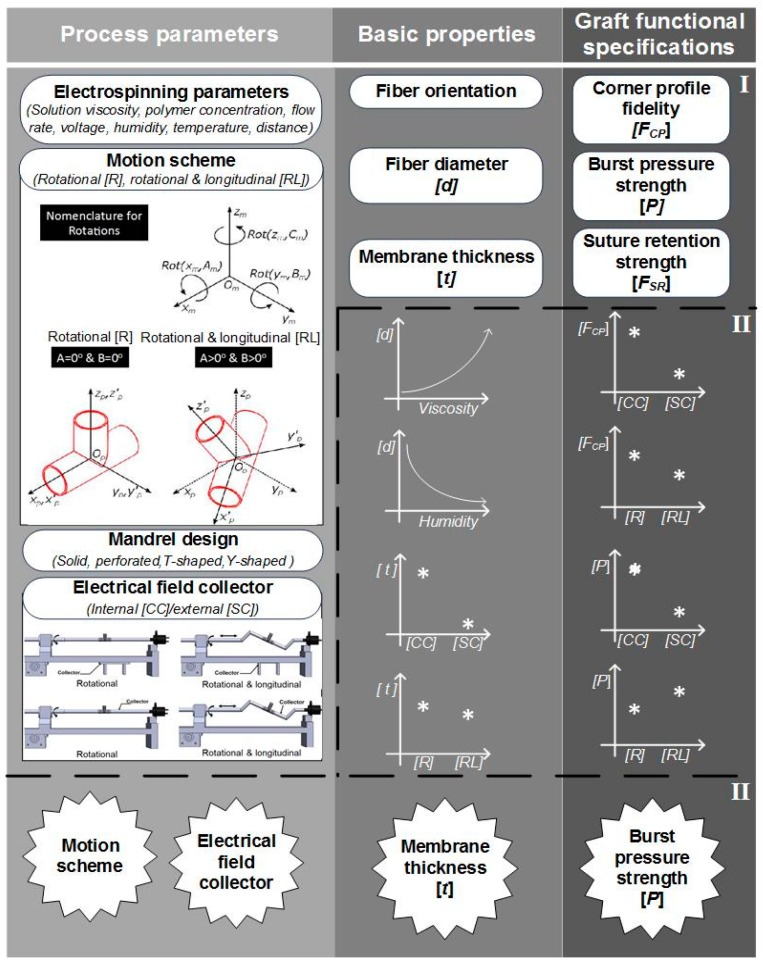
Schematic description of the construction process of bifurcated vascular grafts proposed in this work. (I) Process parameters and properties involved on the construction process’ (II) influence of some process parameters on the basic properties of the graft; III) classification of the most influencing parameters and properties into the graft functional specifications.

In the context of Y-grafts, we identified the following major functional specifications: (a) corner profile fidelity, (b) burst pressure strength, and (c) suture retention strength. These functional specifications correlated to the basic properties of the electrospun fibers and their corresponding membranes, which were from the complete array of process parameters. Figure 22 shows a schematic relationship between the process parameters, basic properties (fibers and membranes) and graft functional specifications. This study reported the significant influence of the internal electrical field collector design and the motion scheme. As we continue to refine this complex fabrication method, a more detailed study of process parameters will be needed (for example: solution viscosity, mandrel design, positioning angles and speed). We also expect to explore variants of this process, such as melt electrospinning and melt electrowriting.

## 5. Conclusions and Future Work

This study aimed to characterize the basic impact of spinning one versus two long axes of vascular graft membrane thickness and the resulting properties of the electrospun vascular graft membranes (see Table 4). These vascular graft membranes were generated by electrospinning PCL solution onto mandrels with bifurcated shapes prepared by 3D printing with E-Shell^®^ 200 (EnvisionTEC, Dearborn, MI) photocurable resin. The values obtained for bifurcated vascular graft membrane thickness and fiber diameter with rotational scheme motion (BLSP_CC_R), 171.22 ± 26.09µm and 276.8 ± 78.5nm respectively, and rotational and longitudinal scheme motion (BLSP_CC_RL), 119.58 ± 24.74 µm and 290.2 ± 104.6nm respectively, were comparable to similar studies with tubular vascular graft membranes (see Table 1). In terms of mechanical properties, the burst pressure strength obtained with rotational scheme motion and rotational and longitudinal scheme motion, 4,388.3 ± 1,027.2 mmHg and 5,126.6 ± 1,074.6 mmHg respectively, were also comparable to tubular vascular graft membranes. The only limitation we have found so far was in terms of suture retention strength, in which the target was set to 2 N and approximately 50% of the expected yield was obtained. Further investigation is required to improve the mechanical performance of bifurcated vascular graft membranes based on refinement of the positioning mechanism kinematics and optimization of process parameters. The first refinement in positioning will be to better ensure orthogonality of the mandrel surface and polymer-exuding needle, followed by control of the rate of spinning. Once membrane apposition to the mandrel is improved, we will be in a position to study the effect of membrane biomaterials. 

In this study we identified the potential applications of bifurcated constructs such as vascular grafts for use in the Fontan procedure (see Figure 1), or pancreatic stents to be used in pancreaticoduodenectomy (PD) procedures (see Figure 2). The scope of this work was limited to successfully obtaining the basic requirements: appropriate morphology and suitable mechanical properties. In the next phase, we expect to continue the development of these devices with additional in vivo and in vitro testing.

## Figures and Tables

**Table 1:** Summary of morphology and mechanical properties of electrospun vascular grafts based on poly(ε-caprolactone) PCL and related materials.

	Morphology		Mechanical Properties		Reference
	Vascular Graft Membrane Shape	Membrane Thickness [Fiber Diameter]	Suture Retention Strength	Burst Pressure	
PCL	Tubular	1.18 ± 0.08mm [150 ± 62 nm]	N/A	2925 ± 600 mmHg	[17]
PCL (12% PCL, 10 mL/h)	Tubular	0.72 ± 0.07 mm [1.5 ± 0.2 μm]	20 N	N/A	[18]
PCL (10% PCL, 3.5 mL/h)		0.91 ± 0.05 mm [1.0 ± 0.1 μm]			
PU	Tubular	295 ± 42 μm [300–600 nm]	7.1 ± 1.2 N	985 ± 75 mmHg	[19]
PCL-PU-LT		615 ± 35 μm	36.2 ± 2.0 N	3580 ± 125 mmHg	
PCL-PU-HT		910 ± 25 μm	45.4 ± 2.6 N	4320 ± 115 mmHg	
PCL	Tubular	[<1μm]	N/A	1550 ± 7.5 mmHg	[20]
PCL/Heparin		[3.5μm]		1560 ± 15 mmHg	
PCL	Tubular	0.4 μm [300–500 nm]	N/A	N/A	[21]
PLA/PCL	Tubular	600 μm [600 ± 400 nm]	N/A	N/A	[22]
PCL–collagen	Tubular	N/A [520 ± 14 nm]	3.0 ± 1.1 N	4915 ± 155 mmHg	[23]
PLA/PLCL	Tubular	0.33 ± 0.03 mm [N/A]	20 N	15,000–22,500 mmHg	[24]

**Table 2:** Characteristics used for the electrospinning experiments.

Factors		Experimental Group Name
Collector	Motion Scheme	
External	Rotational	BLSP_SC_R
External	Rotational and Longitudinal	BLSP_SC_RL
Internal	Rotational	BLSP_CC_R
Internal	Rotational and Longitudinal	BLSP_CC_RL

**Table 3:** Average membrane thickness at different regions.

Experimental Group	Average Thickness per Region [t_R_] (μm)					
	t_R1_	t_R2_	t_R3_	t_R4_	t_R5_	t_R6_
Internal Collector with Rotational Motion (BLSP_CC_R)	140.54 ± 43.2	133.83 ± 46.83	142.21 ± 42.24	169.21 ± 41.78	92.13 ± 24.12	91.42 ± 23.08
Internal Collector with Rotational and Longitudinal Motion (BLSP_CC_RL)	113.29 ± 34.2	135.58 ± 36.54	118.5 ± 31.62	106.46 ± 33.64	67.67 ± 19.9	66.38 ± 23.94

**Table 4:** Summary of morphology and mechanical properties.

Motion Scheme	Corner Profile Fidelity F_CP_ [%] +++		Fiber Diameter *d* [nm]	Membrane Thickness *t* [µm] +++	Burst Pressure Strength *P* [mmHg] +++	Suture Retention Strength *F_SR_* [N]
	R5	R6				
Internal Collector with Rotational Motion (BLSP_CC_R)	33.22 ± 0.03	31.6 ± 0.05	276.8 ± 78.5	171.22 ± 26.09	4388.3 ± 1,027.2	1.04 ± 0.3
Internal Collector with Rot. and Long. Motion (BLSP_CC_RL)	24.23 ± 0.03	25.89 ± 0.05	290.2 ± 104.6	119.58 ± 24.74	5126.6 ± 1074.6	0.83 ± 0.1

NOTE: +++ indicates response variables with statistically significant difference due to motion scheme.
